# Oxidative Stability of Cottonseed Butter Products under Accelerated Storage Conditions

**DOI:** 10.3390/molecules28041599

**Published:** 2023-02-07

**Authors:** Zhongqi He, Sunghyun Nam, K. Thomas Klasson

**Affiliations:** USDA-ARS, Southern Regional Research Center, 1100 Allen Toussaint Blvd., New Orleans, LA 70124, USA

**Keywords:** p-anisidine value, cottonseed, Fourier-transform infrared spectroscopy, peroxide value, tocopherol

## Abstract

Cottonseed is a natural product of cotton (*Gossypium* spp.) crops. This work evaluated the oxidative stability of cottonseed butters through accelerated autoxidation by storage at 60 °C for 25 days. Three oxidative stability parameter values (peroxide value, p-anisidine value, and total oxidation value) were monitored over the storage time. These chemical measurements revealed that the storage stability of the butter products was dominated by primary oxidation of lipid (oil) components, while the secondary oxidation levels were relatively unchanged over the storage time. An analysis of the tocopherols (natural oxidants in cottonseed) suggested not only the protection function of the molecules against oxidation of the cottonseed butter during storage, but also the dynamic mechanism against the primary oxidation of lipid components. Attenuated total reflectance–Fourier-transform infrared spectroscopy (ATR–FTIR) data confirmed no changes in the major C functional groups of cottonseed butters over the storage time. On the other hand, characteristic minor peaks of conjugated dienes and trienes related to lipid oxidation were impacted by the accelerated storage. As each day of accelerated oxidation at 60 °C is equivalent to 16 days of storage at 20 °C, observations in this work should have reflected the oxidative stability behaviors of the cottonseed butters after about 13 months of shelf storage under ambient storage conditions. Thus, these data that were collected under the accelerated oxidation testing would be useful not only to create a better understanding of the autooxidation mechanism of lipid molecules in cottonseed butters, but also in developing or recommending appropriate storage conditions for cottonseed end products to prevent them from quality degradation.

## 1. Introduction

Cottonseed is a natural product of cotton (*Gossypium* spp.) crop. Traditional cottonseed is not suitable for human consumption because of the presence of a toxic ingredient (i.e., gossypol) in its pigment glands [1,2]. Glandless (Gl) cotton is a new variety with gossypol content in its cottonseed <450 mg kg^−1^ dry mass to meet the US FDA’s criteria of gossypol limit as human food [3,4]. Thus, exploration of these Gl cottonseeds as food products would greatly enhance the economic impacts of these new cotton lines [3,5,6]. In addition to rich oil and protein [7], Gl kernels contain some bioactive ingredients and antioxidant activities [8,9,10]. Thus, Gl cottonseeds are promising materials to be processed for the development of nutritional, functional, and bioactive cottonseed food products and/or additives [10,11,12].

In a previous work, Gl cottonseed kernels were formulated to produce a peanut-butter-like product, as plant-based (nut and seed) butters have steadily increased in consumer popularity [13]. Due to the presence of a high oil content, plant butter products are susceptible to autoxidation [14]. The lipid oxidation produces undesirable flavors and aromas and compromises the nutritional quality of fats and oils, leading to the production of toxic compounds [14]. Thus, the oxidative stability is one of the important parameters in consideration in plant-butter storage and improvement [15,16]. The oxidative stability can be evaluated by chemical indicators and spectroscopic analysis [17,18,19]. The peroxide value (PV) and p-anisidine values (AVs) [20] are common chemical indicators used to estimate primary and secondary oxidation products. Attenuated total reflectance–Fourier-transform infrared spectroscopy (ATR–FTIR) has been used to monitor the main structural changes as a consequence of the oxidative treatment in nut and seed products [21].

As a continuation of our effort to enhance the utilization of end-products of cotton as food products, this work monitored the changes in the oxidative stability parameters of cottonseed butter products under accelerated storage conditions (i.e., at 60 °C for 25 days). The objective of this work was to assess and compare the oxidative stability of two cottonseed butter products made from Gl kernels roasted at 150 °C for 15 and 30 min, respectively. Information derived from this work is useful in developing appropriate storage conditions of cottonseed end products to prevent them from degradative auto-oxidation.

## 2. Results and Discussion

### 2.1. Color Profile of Cottonseed Butter

A quantitative measurement of color changes over the storage period was presented in Table 1. The a* values (amount of red (+a*) or green (−a*)) for all samples were positive, ranging from 3.19 to 4.44 for B15 and 3.60 to 4.52 for B30. Compared to the day 0 sample, the hue parameters steadily increased during storage, but no apparent impact trend was observed with the storage time. The b* parameter (i.e., the amount of yellow (+b*) or blue tone (−b*)) was also positive, with greater values (24.68 to 26.73 for B15 and 25.08 to 27.14 for B30), meaning that there was a dominating yellow hue in these samples. Compared to the day 0 sample, the hue parameter decreased slightly during the early storage time (6–10 days); it then increased steadily and slowly with a longer storage time. On the other hand, the comprehensive lightness (L* values) decreased slightly over the storage time, with one exception (B30 day 20). The color-profile changes in the two cottonseed butter products were similar to those in pistachio paste and spreads during similar accelerated storage for 25 days at 60 °C [16]. The color-profile parameters of sesame paste products also fluctuated slightly during the whole storage process for 180 days, no matter the storage temperature, at 4 °C, room temperature (average 20.5 °C), or 40 °C [17].

The derived parameters of butter color, color saturation (C*), and color hue (h) were also impacted slightly by storage. Color saturation is a measure of color purity, while the color hue represents the degree of green color. As the set of color-purity data (C*) changed in the similar way to that of L* and b* in the storage butter samples, we could conclude that the accelerated storage did not dramatically impact the homogeneity and uniqueness of the cottonseed butter products regarding t0he coloration. The values of color hue (h) decreased with the longer storage, a reverse trend of the change of the a* parameter. This observation suggested some blanching effect with a longer storage time. In addition, it is noticeable that the L* values of B15 samples were higher than the corresponding B30 samples, while the C* values of the former were lower than the latter. This observation was apparently due to the darkening effect of the longer roasting time (30 min vs. 15 min) during the kernel roasting.

### 2.2. Impact of Accelerated Storage on the Oxidative Stability Parameters

**Figure 1:**
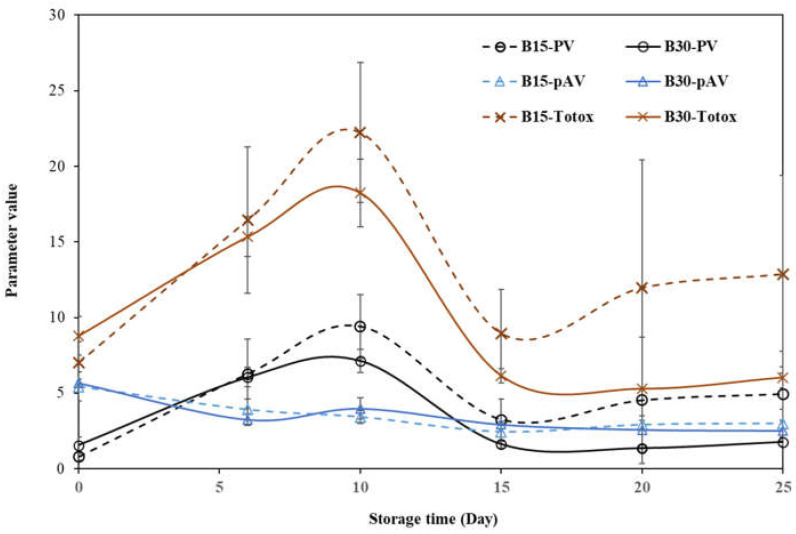
Changes in the oxidative stability values of cottonseed butters over the accelerated oxidation storage at 60 °C. B15 and B30, butter products made from the cottonseed roasted at 150 °C for 15 and 30 min, respectively. PV, peroxide value; pAV, p-anisidine value; Totox, total oxidation value. Data are present in the format of average ± standard deviation (n = 3).

Figure 1 shows the changes in the three sets of oxidative stability parameters measured in this work over the accelerated auto-oxidation experiment at 60 °C. Generally, the impact trends on the parameters were similar between B15 and B30, indicating the consistence of these observations in butter products with different roasting times. However, these values of B15 were slightly lower than B30 on day 0, but they gradually became higher than B30 samples with the same storage times. This result implied that the butter product with 30-min roasting was more resistant to autoxidation during storage, although the cottonseed kernels could have been subject to a little more lipid oxidation with the longer roasting.

The peroxide value is a measure of the concentration of peroxides and hydroperoxides formed in the initial stages of lipid oxidation so that it is one of the most widely used tests for the measurement of oxidative status in seed and nut products [15]. In this work, we observed that the value of both the B15 and B30 samples rapidly increased in the first 10 days of the storage at 60 °C and then decreased and slowly increased afterward. Generally, the longer the storage time, the higher the peroxide values [17,22]. Thus, the fluctuation we observed might reflect the fact that hydroperoxides in the cottonseed butter samples were unstable at a high temperature, and some of them started to decompose into carbonyl compounds, aldehydes, and other secondary oxidations [23,24]. Another fact was that the oil constituent in the cottonseed butter products was from the cottonseed kernels and the extra cottonseed oil added. The indigenous oil part might have been more tightly associated with other kernel components. Thus, this sort of inhomogeneity might also partially contribute to the fluctuation of peroxide value curves and other observations reported later in this work. In addition, it is assumed that naturally present oxidant tocopherol in pistachio spreads delayed lipid oxidation so that no substantial differences were observed between those spreads during storage for 25 days at 60 °C [16]. Generally, maximum peroxide values of 10–30 milliequivalents of peroxide per kg (meq kg^−1^) are acceptable dependent on a specific product. For example, 30 meq kg^−1^ was recommended as the limit of sunflower paste [15]. To our knowledge, there are no data on the peroxide values of cottonseed products for comparison. However, the values of B15 and B30 between 0.79 and 9.41 meq kg^−1^ were lower than those values (6–18 meq kg^−1^) of fresh produced sunflower butter from pan-roasted kernels during 60 days of storage [25]. It is reported that each day of accelerated oxidation at 60 °C is equivalent to 16 days of storage at 20 °C [24]. Thus, our data should have reflected the oxidative stability behaviors of the two cottonseed butter products with about 13 months of shelf storage under ambient storage conditions.

The p-anisidine value is another oxidative-stability parameter. It represents the decomposition of the hydroperoxides in oil products. The p-anisidine values of B15 and B30 were around 5.5 at the beginning of the storage experiment. The values were lower in the stored samples, but they seemed not to change much over the storage time. Lower and fluctuating p-anisidine values over storage were also observed with sunflower paste [15], sesame paste [17], and sunflower and olive oils at ambient temperatures [20]. While it is used as an empirical test for monitoring the secondary products of lipid oxidation [16], as a rule of thumb, this parameter should be less than 10 for a good-quality oil [26]. Thus, the p-anisidine values of both B15 and B30 met the quality requirement well.

Total oxidation (Totox), a combination of both peroxide values and p-anisidine value, is considered to be an overall assessment of the oil oxidation process, so that its value is used as an indication of the overall oxidative stability, including both primary and secondary oxidation products [22]. In other words, the Totox values reveal the past history of the oil oxidation (p-anisidine value) and its present status (peroxide value) [16]. The Totox change pattern of B15 and B30 over the accelerated storage was similar to that of the peroxide values, as the peroxide values were higher than the p-anisidine values in B15 and B30 samples. Therefore, the impacts of the storage on the lipid oxidation (deterioration) of the two cottonseed butter products seemed a more dynamic (present state) process than an accumulative (past history) process.

### 2.3. Tocopherol Contents in Cottonseed Butter and Impact of Storage Times

Since it is present in greater concentrations in cottonseed oil compared to other vegetable oils, tocopherol has been reported to be the main reason for oxidative stability and prolonged shelf life of cottonseed oil [27]. Under the current analysis conditions, the applied HPLC method detected quantitatively four types of tocopherols in the cottonseed butter samples (Table 2). The total tocopherol contents were between 45 to 107 mg kg^−1^. The tocopherol contents in the B15 series were generally lower than those in the corresponding B30 samples. This is because the low temperature and short roasting time are not always the most appropriate ways to obtain the best product in terms of total phenolic content, tocopherols, and volatile profile [28,29]. The contents of a- and γ-types ranged from 10 to 45 and 29 to 67 mg kg^−1^ in the B15 and B30 butter samples, respectively. The contents of the other two types, d- and b-tocopherol, were much lower, accounting for 0.8–3.5% of total tocopherol content in B15 and B30. Kouser, et al. [30] reported a-tocopherol (125–296 mg kg^−1^), γ-tocopherol (269–326 mg kg^−1^), and d-tocopherol (2.23–5.47 mg kg^−1^) among cottonseed oils from six varieties of cotton, while no data for b-tocopherol were reported. Considering only 43% of the butter samples as the oil content, our data were at similar levels to the literature data of tocopherol in cottonseed. Thus, the high tocopherol content benefited cottonseed butter’s prolonged shelf life [16] and promoted vitamin E activity [31]. Javidipour, et al. [32] also reported a-tocopherol content at the same level. However, the content of γ-tocopherol (42–73 mg kg^−1^ of oil) in their cottonseed oil was lower. The difference is probably due to the fact that the sample used by Javidipour, Tüfenk and Baştürk [32] was the commercial cottonseed oil product purchased from local supermarkets. Indeed, as high as 1000 mg kg^−1^ of tocopherols could be in unprocessed cottonseed oil, but up to one-third can be lost during processing (e.g., deodorization) [33]. In this work, part of tocopherol content of the butter samples should have been from the indigenous cottonseed kernels, and part from the external cottonseed oil. In this work, we observed a general decreasing trend of the total tocopherol content in the samples after storage at 60 °C; however, the differences were not statistically significant at *p* = 0.05. This phenomenon might reflect the protection function of the consuming tocopherol against oxidation of the cottonseed butter during storage. In addition, those high standard deviations with the poor parallelism may also reflect the fact that there were some minor differences in the treatment and analysis of these replicate samples, as they were susceptible to autoxidation under aerobic circumstances.

To gain more insight into these tocopherol contents and their roles in oxidative stability, a correlation analysis was performed to elucidate the quantitative correlation coefficients between those parameters (Table 3). Three types of tocopherols (i.e., a-, γ-, and δ-tocopherol), as well as total tocopherol content, were well correlated with each other, with *p* ≤ 0.01. However, β-tocopherol did not show any statistically significant (*p* > 0.05) correlation coefficient with other tocopherol contents or oxidative stability. While b-tocopherol showed no significant (*p* > 0.05) coefficient with oxidative stability, the other four tocopherol contents also showed statistically significantly (*p* ≤ 0.05) but negative correlation coefficients with two of the three oxidative stability parameters (i.e., the peroxide values and Totox values). On the other hand, there was no statistically significant (*p* < 0.05) coefficient between the p-anisidine value and any type of tocopherol content. A negative correlation between the peroxide value and total tocopherol content is also observed in roasted peanuts during accelerated storage [34]. Those outcomes confirmed not only the positive impact of the major types of tocopherols on the oxidative stability of the cottonseed butter during storage and processing [30], but also the dynamic mechanism against the primary oxidation (present state).

### 2.4. ATR–FTIR Observations

**Figure 2:**
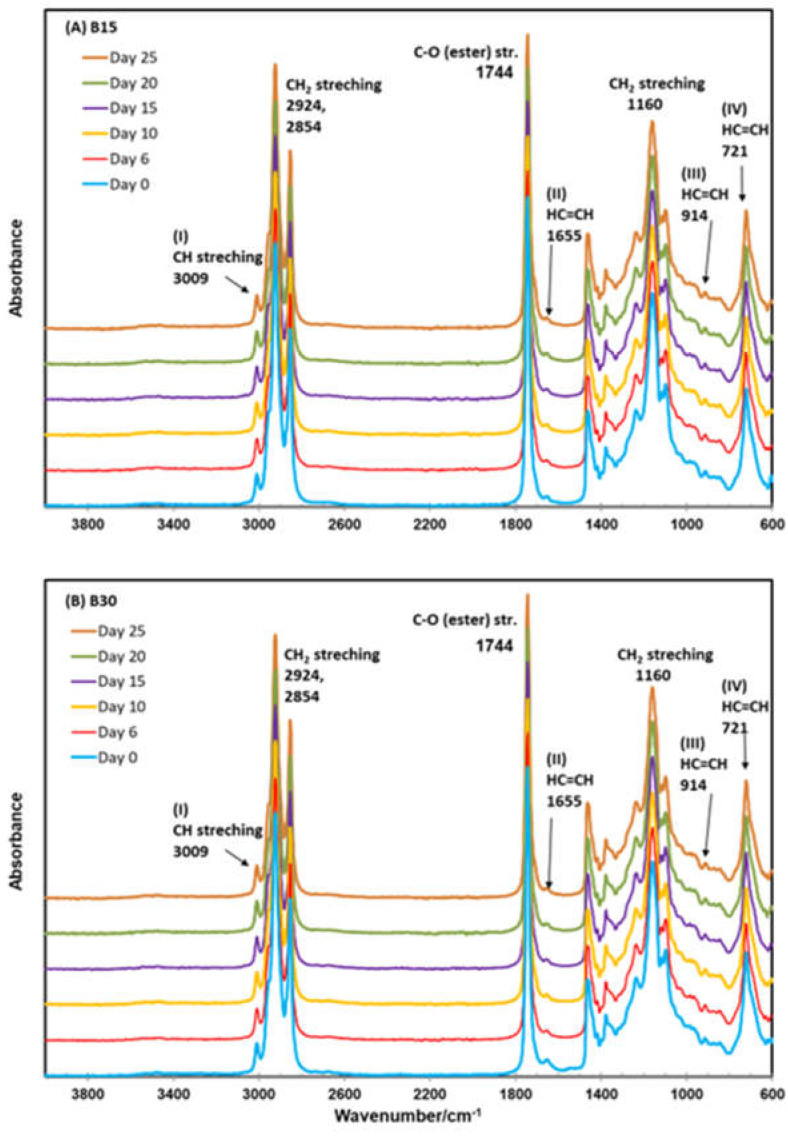
ATR–FTIR spectra of oil extracts of cottonseed butters over the accelerated oxidation storage for 25 days at 60 °C. (**A**) B15 and (**B**) B30 are butter products made from the cottonseed roasted at 150 °C for 15 and 30 min, respectively.

ATR–FTIR analysis has been used in cotton biomass characterization [35,36,37]. The ATR–FTIR spectra of the oil extracts of the cottonseed butters (Figure 2) were typical to those of cottonseed and other vegetable oil samples in the literature [7,35,38]. The four major peaks were found at 2924, 2854, 1744, and 1160 cm^−1^. The first two peaks could be assigned to the asymmetric and symmetric stretching vibration of the C–H bond in the aliphatic –CH_3_ group. The third peak was due to the easter carbonyl functional group of triacylglycerides. The last peak and the flanking minor 1236 and 1099 cm^−1^ peaks could come from the stretching and rocking vibration of the –C–O ester group and –CH_2_– group. The overall appearance of the ATR–FTIR spectra of the oil extracts of 12 cottonseed butters over the accelerated oxidation storage for 25 days at 60 °C was very similar, implying that the backbone C-functional groups (i.e., the major components of oil fractions) did not change over the accelerated storage period. This observation was consistent with previous reports on FTIR investigations of oxidative stability of edible oil products [39,40,41].

**Figure 3:**
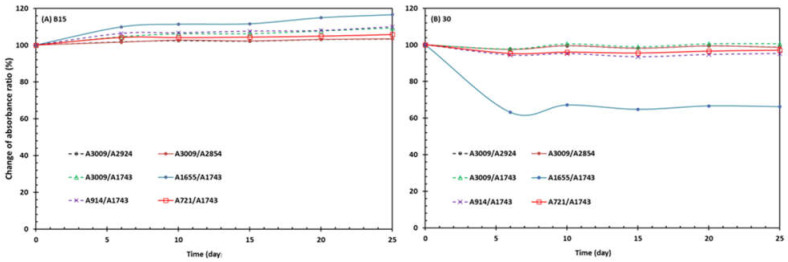
Relative changes (%) of selected ATR–FTIR absorbance ratios over the accelerated oxidation storage for 60 days at 60 °C. (**A**) B15 and (**B**) B30 are butter products made from the cottonseed roasted at 150 °C for 15 and 30 min, respectively.

The impacts of oxidative stability on FTIR features were found to be more on the characteristic minor peaks of conjugated dienes and trienes [41,42,43]. In this work, we selected the four peaks at 3009, 1655, 914, and 721 (labeled as I–IV in Figure 2) for our evaluation of the FTIR changes over the accelerated storage. The peak at 3009 cm^−1^ was associated with the stretching vibration of the CH cis-olefinic group (=C-H), and its intensity decreased with the increasing degrees of oxidation. The peaks at 1655, 914, and 723 cm^−1^ reflected the changes in double bonds (–HC=CH–) during the oxidation of oil samples. In other words, their absorbance corresponded to the oxidation products formed by polyunsaturated fatty acids, such as conjugated aldehydes and the conjugated double-bond system [40,41]. The relative changes in their absorbance ratios relative to the major peaks at 2924, 2854, or 1774 cm^−1^ are shown in Figure 3. At the beginning of the storage (day 0), the ratios of A3009/A2924, A3009/A2854, and A3009/A1744 were 0.125, 0.185, and 111 for B15 and 0.128, 0.189, and 0.109 for B30, respectively. These values were comparable but a little lower than the values (0.164, 0.211, and 0.137) of untreated sunflower oil samples in the literature [43]. The three ratios of B50 samples increased steadily with storage time, although the increasing percentages were small (3.35%, 3.43%, and 9.31%, respectively, at day 25). On the other hand, storage over 25 days at 60 °C did not change or slightly lower the three ratios of B30 samples, with the changes by −1.18%, −1.40%, and 0.67, respectively, at the end of storage. The changes in these ratios were much smaller compared to those (27–40% increases) of sunflower oil heated at 183 °C for 8 h [43]. While addition of antioxidant grape pomace extracts can inhibit the thermo-oxidative degradation of sunflowers [43], the indigenous antioxidants tocopherol (Table 2), peptides [10,44], and other bioactive compounds in cottonseed [29] should have reduced the auto-oxidation of cottonseed butter so that the smaller changes in the FTIR absorbance ratios were observed.

The change trends of A914/A1744 and A721/A1744) were similar to the three with A3009, as discussed above. Another ratio (A1655/A7144) showed greater changes. Compared to the other five ratios, its percentage change increased more in B15 samples after storage, with an average of 16.58%, and it also decreased more (−33.79%) in B30 samples. Previously, Rohman and Che-Man [42] observed that the peak intensities at 1655 and 967 cm^−1^ increased, but the peak intensities at 3008 and 722 cm^−1^ decreased during the thermal oxidation of vegetable oils. In a closely examined study, Wen et al. [41] reported that both peaks at 914 and 723 cm^−1^ of walnut oil samples exhibited a downward trend over the 7-day period of storage at 60 °C. However, the peak at 1654 cm^−1^ of these walnut oil samples initially decreased, then increased, and decreased again over storage. Wen et al. [41] attributed the complicated pattern to the continuous degradation of *cis* double bonds (olefinic group) at the initial oxidation stage. In the meantime, secondary oxidation products started to be produced with the deepening oxidation, reflecting the increasing absorbance at 1654 cm^−1^. Despite this, the degradation of the double bond kept playing great roles, reversing the A1654 change to a downward trend at the ending phase of the storage. This mechanism may be applicable to the different observations of these cottonseed samples, as the secondary oxidation parameter, p-anisidine value, was a little lower, but the primary oxidation parameter, peroxide value, was higher over the storage period at 60 °C (Figure 1). The FTIR difference between B15 and B30 implied the double functions of longer roasting time of speeding auto-oxidation and activating more antioxidant ability, including tocopherol (Table 2). However, the correlation analysis found only one significant correlation coefficient (0.721, *p* < 0.01) between A3009/A1744 and b-tocopherol. All other correlation coefficients between the six FTIR absorbance ratios and seven chemical measurements were not statistically significant (*p* > 0.05). Therefore, more research is needed to better understand the changes in the FTIR features of cottonseed butter and other products during auto-oxidation and food stability.

## 3. Materials and Methods

### 3.1. Cottonseed Source and Peanut-Butter-like Product Making

The Gl cottonseed of NuMex series was provided by Cotton, Inc. (Cary, NC, USA) [5,45]. These seeds were dehulled mechanically by cracking with a 20.32 cm plate mill and then separated with a vibratory shaker. The kernel products were further cleaned by passing the material through a laboratory aspirator to remove the non-kernel material [7]. Cottonseed oil (Admiration Foods, Englewood, NJ, USA) was acquired from a local store.

The peanut-butter-like products were made based on the procedure reported previously [13]. Gl cottonseed kernels were used as the base material (75.0% of total product) to produce peanut-butter-like spread formulations. Additional cottonseed oil (16.8%), cane sugar (7.5%), and table salt (0.7%) were the other three ingredients used in the formulation. The basic composition of the formulation is listed in Table 4. For butter making, the Gl cottonseed kernels (240 g) in a metal tray were first roasted in a convection oven (Thermo Scientific Precision Com-pact Ovens, Waltham, MA, USA) at 150 °C for 15 or 30 min. After they were cooled down, the roasted kernels were ground with Waring Commercial Blender (Model WF2211214, Torrington, CT, USA) at a high speed for 3 min; they were then mixed thoroughly with the other three ingredients by a spatula. The mixture was then passed through a Smokehouse meat grinder with a 4 mm hole plate (Buchanan Dam, TX, USA). The two extrudants with kernels roasted for 15 and 30 min were taken as the peanut butter products (i.e., cottonseed butters), and named B15 and B30, respectively.

### 3.2. Accelerated Storage Experiment

This accelerated storage procedure was based on the literature on the evaluation of oxidative stability of plant butters [16,17]. The cottonseed butter samples (11.00 g each) were weighed into separate 50 mL centrifuge tubes. These were then capped and placed in a preheated (60 °C) forced-air convection oven (Thermo Scientific, Waltham, MA, USA) for up to 25 days. The interior of the oven remained dark throughout the storage period. Triplicate tubes were removed after 0, 5, 10, 15, 20, and 25 days, respectively.

### 3.3. Cottonseed Color Determination

The color of the butter samples was analyzed with a Spectro2guide spectrophotometer with built-in calibration standard in the docking station (BYK-Gardner, Columbia, MD, USA) and recorded on the CIE L*a*b* color coordinates [46]. The axis L* evaluated a sample from black to white; the axis a* from green to red; and the axis b* from blue to yellow. In addition, two derived values, the color hue (h) and color saturation (C*) of the samples, were calculated from values of a* and b* [46,47].

### 3.4. Extraction of Oil Fractions from Butter Products

Oil fractions of the butter samples were extracted by petroleum ether via a Soxhlet distillation apparatus (ST 243 Soxtec solvent system, FOSS Analytics, Hillerod, Denmark) [17,48]. The extraction temperature and time parameters were 60 °C and 4.5 h, respectively. The extracted oil fractions were then placed under a fume hood overnight to let the solvent evaporate out. These oil samples were kept in a freezer (−20 °C) until analysis.

### 3.5. Oxidative Stability Measurement

Oxidative stability parameters were measured from the oil fractions of the cottonseed butter samples incubated over the accelerated autoxidation period [22]. Prior to analysis, the oil samples were removed from the freezer and allowed to equilibrate to room temperature [16]. The peroxide value (*PV*) was then determined per the protocol of AOCS Official Method Cd 8b-90 [49]. Data were expressed as milliequivalents of peroxide per kg of butter sample (meq kg^−1^) [50]. The p-anisidine value (*p-AV*) was determined according to the standard of AOCS Official Method Cd 18-90, using a spectrophotometer at 350 nm [51]. The total oxidation (*TOTOX*) *value* [22,52], as a useful measure of initial degradation in the oil that provides good information on both primary and secondary products of oxidation, was calculated as follows:

*TOTOX value* = *2PV* + *p-AV* (1)

### 3.6. HPLC Determination of Tocopherol

The analysis of tocopherol was conducted per the AOCS procedure Ce 8-89 [53]. Each oil extract (20 mg) was dissolved in 1.0 mL of hexane. The measurement was conducted with a Waters (Milford, MA, USA) model 2695 HPLC pumping system, a model 2475 fluorescence detector, and a Phenomenex Luna 5 μm Silica(2) column (150 mm × 4.6 mm). The mobile phase was 99% hexane and 1% isopropanol, with a pumping rate of 1.0 mL min^−1^. The injection volume was 20 µL. Tocopherols were detected at an excitation wavelength of 290 nm and an emission wavelength of 330 nm. Concentrations were determined by relating sample peak areas to standard curves and converted to mg kg^−1^ of butter sample.

### 3.7. ATR–FTIR Spectroscopy

The C functional group analysis of the oil fractions of the butter samples was conducted by a Vertex 70v FTIR spectrometer (Bruker Daltonics, Billerica, MA, USA), which was equipped with a MIRacle ATR accessory (Pike Technologies, Fitchburg, WI, USA) that incorporated a diamond crystal plate as the reflector. A few drops of the oil samples were placed on the ATR crystal surface and secured with a metal clamp to ensure a reproducible pressure, which was applied to the samples to achieve intimate contact with the ATR crystal. The spectra were collected over the range of 4000–600 cm^−1^, at a 4 cm^−1^ resolution and with 16 scans. After each measurement, the ATR plate was carefully cleaned by wiping it with analytical-grade acetone and dried with a soft tissue before it was filled with the next sample [39]. All spectra were normalized and presented in absorbance [36].

### 3.8. Statistical Analysis

Triplicate data of the color and chemical analysis are presented in the format of average ± standard deviation. These average values, if significantly different at *p* ≤ 0.05, are indicated by different letters in Table 1 and Table 2. The Correlation Analysis Tool under Data Analysis in Microsoft Excel 2016 was used to analyze correlation coefficients between the data sets of different parameters.

## 4. Conclusions

In this work, two cottonseed butter products (B15 and B30) were made from glandless cottonseed kernels roasted at 150 °C for 15 and 30 min, respectively. Their oxidative stability was evaluated by an accelerated storage experiment at 60 °C for 25 days. The colorimetric profile data indicated that the accelerated storage did not dramatically impact the color homogeneity and uniqueness of the cottonseed butter products, while there was a slight blanching effect with longer storage time. The measurements of three oxidative stability values revealed that the storage stability of the butter products was dominated by primary oxidation of lipid (oil) oxidation, while the secondary oxidation products were relatively unchanged over the storage time. High levels of antioxidant tocopherols detected in the cottonseed butter reflected the protection function of the tocopherol against oxidation of the cottonseed butter during storage. The ATR–FTIR data confirmed no changes in the major components of cottonseed butters over the storage time, but they indicated changes in double bonds related to lipid oxidation impacted by accelerated storage conditions. Overall, those data collected under the accelerated oxidation testing at 60 °C in 25 days should have reflected the oxidative stability behaviors of the cottonseed butter after about 13 months of shelf storage under ambient storage conditions. While these measured parameters of oxidative stability were critical in regard to food quality and flavors, the impacts of storage on other parameters (e.g., texture and physicochemical quality) should also be evaluated in future work for consumer acceptability of the cottonseed butter products [15,50].

## Figures and Tables

**Table 1:** Changes in the colorimetric profile of cottonseed butters over the accelerated oxidation storage at 60 °C. B15 and B30, butter products made from the cottonseed roasted at 150 °C for 15 and 30 min, respectively. Data are present in the format of average ± standard deviation (n = 3). Different letters in the same column indicate these values are statistically significantly different (*p* ≤ 0.05).

(A) B15					
Day	L*	a*	b*	C*	h
0	59.52 ± 0.01 a	3.19 ± 0.02 f	25.60 ± 0.04 d	25.80 ± 0.04 e	82.89 ± 0.05 a
6	55.21 ± 0.02 f	4.08 ± 0.01 d	24.68 ± 0.01 f	25.01 ± 0.01 f	80.61 ± 0.02 e
10	55.30 ± 0.02 e	4.44 ± 0.01 a	25.40 ± 0.01 e	25.87 ± 0.01 d	80.11 ± 0.01 f
15	57.67 ± 0.02 d	4.21 ± 0.01 b	26.42 ± 0.01 b	26.75 ± 0.01 bc	80.94 ± 0.01 d
20	59.01 ± 0.01 b	3.99 ± 0.01 e	26.11 ± 0.01 c	26.41 ± 0.01 c	81.32 ± 0.01 b
25	58.54 ± 0.00 c	4.18 ± 0.00 c	26.73 ± 0.00 a	27.05 ± 0.01 a	81.12 ± 0.01 c
**(B) B30**					
**Day**	L*	a*	b*	C*	**h**
0	56.58 ± 0.01 b	3.60 ± 0.01 f	26.15 ± 0.04 d	26.41 ± 0.01 d	82.16 ± 0.00 a
6	56.35 ± 0.01 d	4.30 ± 0.01 d	26.06 ± 0.00 e	26.42 ± 0.01 d	80.62 ± 0.00 e
10	55.47 ± 0.05 e	4.52 ± 0.01 a	25.68 ± 0.01 f	26.08 ± 0.00 e	80.01 ± 0.02 f
15	54.78 ± 0.01 f	4.04 ± 0.01 e	26.37± 0.01 c	26.68± 0.01 cc	81.28 ± 0.00 b
20	57.77 ± 0.01 a	4.46 ± 0.01 b	27.14 ± 0.01 a	27.51 ± 0.01 a	80.66 ± 0.02 d
25	56.45 ± 0.01 c	4.34 ± 0.01 c	26.78 ± 0.00 b	27.13 ± 0.01 b	80.79 ± 0.01 c

**Table 2:** Changes in individual and total tocopherol content (mg kg^−1^ of butter sample) of cottonseed butters over the accelerated oxidation storage at 60 °C. B15 and B30 are butter products made from the cottonseed roasted at 150 °C for 15 and 30 min, respectively. Data are presented as average ± SD (n = 3). Same letters in the same column indicate that these values are not statistically significantly different (*p* > 0.05).

(A) B15					
Day	a-	γ-	δ-	b-	Total
0	23.30 ± 10.30 ab	62.28 ± 15.12 a	0.66 ± 0.05 a	0.73 ± 0.27 c	87.18 ± 15.14 a
6	14.23 ± 11.16 ab	43.49 ± 16.81 ab	0.52 ± 0.11 a	1.24 ± 0.37 ab	59.48 ± 28.41 a
10	9.68 ± 8.63 b	34.30 ± 18.15 ab	0.54 ± 0.06 a	1.16 ± 0.29 abc	49.70 ± 31.19 a
15	31.01 ± 8.33 a	61.97 ± 2.82 a	0.69 ± 0.04 a	1.10 ± 0.07 bc	94.78 ± 10.89 a
20	24.27 ± 8.84 ab	52.30 ± 11.81 ab	0.68 ± 0.13 a	0.98 ± 0.27 bc	78.22 ± 20.66
25	15.09 ± 17.66 ab	28.52 ± 27.32 b	0.52 ± 0.15 a	1.58 ± 0.21 a	45.72 ± 44.87 a
**(B) B30**					
**Day**	**a-**	**γ-**	**δ-**	**b-**	**Total**
0	30.28 ± 17.24 b	66.24 ± 1.43 a	0.74 ± 0.06 b	0.77 ± 0.07 bc	98.03 ± 8.71 a
6	21.36 ± 3.39 c	43.27 ± 4.54 b	0.65 ± 0.09 bc	0.80 ± 0.09 b	66.09 ± 7.13 b
10	14.06 ± 5.91 c	29.70 ± 7.79 c	0.57 ± 0.05 c	0.68 ± 0.05 c	45.01 ± 13.55 c
15	45.45 ± 0.81 a	66.85 ± 1.68 a	0.92 ± 0.03 a	1.07 ± 0.01 a	114.29 ± 2.24 a
20	45.33 ± 0.66 a	64.63 ± 0.19 a	0.97 ± 0.04 a	1.10 ± 0.02 a	112.03 ± 0.80 a
25	41.25 ± 2.03 ab	63.35 ± 0.35 a	0.96 ± 0.04 a	1.08 ± 0.03 a	106.64 ± 2.25 a

**Table 3:** Correlation coefficients between tocopherol contents and oxidative stability parameters collected from cottonseed butter samples B15 and B30 over the accelerated oxidation storage at 60 °C (n = 12). PV, peroxide value; pAV, p-anisidine value; Totox, total oxidation value.

		Tocopherol					Oxidative Stability	
		α-	g-	d-	b-	Total	PV	pAV
Tocopherol	g-	0.851 ***						
	d-	0.974 ***	0.802 **					
	b-	−0.069	−0.275	−0.129				
	Total	0.957 ***	0.967 ***	0.916 ***	−0.179			
Oxidative stability	PV	−0.815 ***	−0.873 ***	−0.747 **	0.163	−0.880 ***		
	pAV	−0.316	0.059	−0.314	−0.543	−0.123	−0.121	
	Totox	−0.881 ***	−0.866 ***	−0.812 **	0.06	−0.909 ***	0.981 ***	0.074

Symbols **, and *** indicate the coefficient values significant at *p* = 0.01, and 0.001, respectively.

**Table 4:** Basic composition of cottonseed butter. Values were calculated per the composition data of the cottonseed kernels [7] and percentages of four ingredients (i.e., cottonseed, cottonseed oil, cane sugar, and table salt) in the butter formulation. ADF, acid detergent fiber. ADL, acid detergent lignin.

Major Component (g kg^−1^)						
Protein	Oil	Sugar	Starch	ADF	ADL	Gossypol
315.8	430.5	75.0 ^1^	12.5	81.8	50.9	0.0045
**Macro Element (g kg^−1^)**						
P	Ca	K	Mg	Na	S	Cl
8.6	1.7	9.6	4.6	3.2	3.7	4.2

^1^ For added sugar only.

## Data Availability

The data presented in this study are available wholly within the manuscript.

## Sample Availability

Samples of the butter products are available from the authors upon formal request and cooperative agreement.
